# The readmission of a parturient with natural delivery after discharge due to residual fetal membrane: A case report

**DOI:** 10.1097/MD.0000000000034565

**Published:** 2023-08-11

**Authors:** Yonghong Wang, Juan Li, Qiuyang He, Jianhua Ren, Guoyu Wang

**Affiliations:** a Department of Obstetric Nursing, West China Second University Hospital, Sichuan University/West China School of Nursing, Sichuan University; b Key Laboratory of Birth Defects and Related Diseases of Women and Children (Sichuan University), Ministry of Education, Chengdu, Sichuan, China.

**Keywords:** case report, placenta examination, placental residue

## Abstract

Placental residue is a relatively common and sophisticated disease among obstetric delivery complications. A failure to detect placental residue in time may cause poor outcomes such as postpartum hemorrhage and puerperal infection.

**Patient concerns:**

We present the case of a 33-year-old full-term singleton parturient with placental residue. Upon precipitate labor and childbirth, the placenta and fetal membranes were examined to be intact. However, 1 day after discharge, she felt that there was discharge from the vagina and thus presented to our emergency department.

**Diagnoses:**

The patient was diagnosed with residual membranes and readmitted to the hospital for uterine curettage.

**Intervention:**

Uterine curettage was performed under B-ultrasound guidance.

**Outcome:**

The patient was discharged smoothly without any postoperative complications.

**Lessons:**

This paper can provide significant enlightenment for the prevention and early treatment of placental residue, including enhancing the risk awareness of high-risk patients, standardizing the process of clinical examination of the placenta, and early uterine contraction promotion to assist in the discharge of residual tissue, so as to reduce the occurrence of placental residue.

## 1. Introduction

Placental residue is a relatively common and sophisticated disease among obstetric delivery complications. The incidence rate of placental residue after vaginal delivery is 1% to 3%.^[1]^ A failure to detect placental residue in time may cause serious adverse outcomes such as severe postpartum hemorrhage, uterine rupture, infection, hysterectomy, and even death. With the enactment of the third-child policy in China, the accompanying high-risk factors such as cesarean section and assisted reproductive technology have also increased, which may lead to a further increase in the incidence rate of placenta residue.

## 2. Case report

A 33-year-old parturient (*G2P2*: pregnancy twice, giving birth twice) with intrauterine pregnancy gave birth to a live baby via spontaneous vaginal delivery at 40 + 3 weeks (cephalic presentation). The delivery was a successful precipitate labor for 2 hours and 31 minutes (the third stage: 10 minutes) and the perineal laceration was degree I. The placenta was delivered spontaneously. The placenta and fetal membranes were examined to be intact. There was nothing special during the whole course. The parturient was discharged after receiving postpartum treatment of promoting uterine contraction. One day after discharge, the patient felt that there was discharge from the vagina and thus presented to our emergency department. During examination, the decidua-like tissue prolapsing from the cervical orifice was observed and then clamped. The results of Color Doppler ultrasound showed that there was slightly strong heterogeneous echo with the range of about 5.0 × 2.4 × 3.7 cm in the middle segment of the uterine cavity with unclear boundary. No obvious blood flow signal was detected. The patient was diagnosed with intrauterine occupying lesion (residual membranes) and readmitted to the hospital for further treatment.

On admission, the patient’s temperature was 36.8 °C, pulse rate was 84/min, respiratory rate was 21/min, blood pressure was 109/63 mm Hg, white blood cell count was 11.8 × 10^9^/L, neutrophil count was 6 × 10^9^/L, and hemoglobin was 121 g/L. Uterine curettage was performed under B-ultrasound guidance. During the operation, the uterine was found to be at the horizontal position, with the size similar as the uterine at 50-day pregnancy. Before the operation, the depth of the uterine cavity was detected as 15 cm. Under the guidance of ultrasound, the fetal membranes (2 × 5 cm) and 30 g blood clots were clamped and suctioned out. The morphology of the uterine cavity was relatively regular. After the operation, the depth of the uterine cavity was detected as 15 cm, and the uterus contracted well with less vaginal hemorrhage. The operation was successful, and the patient’s vital signs remained stable during the operation. The patient was intravenously given 10u oxytocin. During uterine curettage, the blood loss was 100 mL, the infusion was 500 mL, and the urine volume was 50 mL. There was no intraoperative complication. After the operation, the patient returned to the ward and was given treatments of oxytocin to promote uterine contraction and antibiotics to prevent infection. The patient’s vital signs and vaginal bleeding were carefully monitored.

## 3. Discussion

At present, placental residue is a relatively common and sophisticated disease among obstetric delivery complications, with an incidence of 1% to 3% after vaginal delivery.^[1]^ There were numerous influencing factors, and the independent risk factors affecting postpartum placental residue, in descending order according to the risk degree, are the third stage of spontaneous delivery, uterine inertia complicated with postpartum hemorrhage ≥ 500 mL, uterine inertia, scarred uterus with vaginal delivery, manual removal of placenta, postpartum injection of oxytocin < 2 d, pregnancy ≤ 2 years after the last pregnancy, abortion history, pregnancy complicated with hypertension, pregnancy complicated with gestational diabetes mellitus, and age ≥ 35 years.^[2]^ The following reports were made based on the retrospective analysis and follow-up results of this case.

Review of the relevant knowledge: The diagnostic criteria of residual placenta are as follows: ① The placenta and membranes are incomplete or surface of placenta is rough after placental delivery in the third stage of delivery; Continuous vaginal bleeding occurs after placental delivery. ② B-ultrasound examination indicates that there is defect on the surface of maternal placenta or fetal membrane; There is uneven and strong echo in uterine cavity with single or multiple hyper-echo masses. ③ After uterine curettage or other medication treatment, a small amount of residual placental tissue or fetal membranes are discharged which are confirmed as postpartum placental residue by pathological examination.^[3,4]^ Therefore, the diagnosis of placental residual requires a combination of factors including medical history, clinical manifestations and imaging examinations.^[2]^

**Figure 1.**
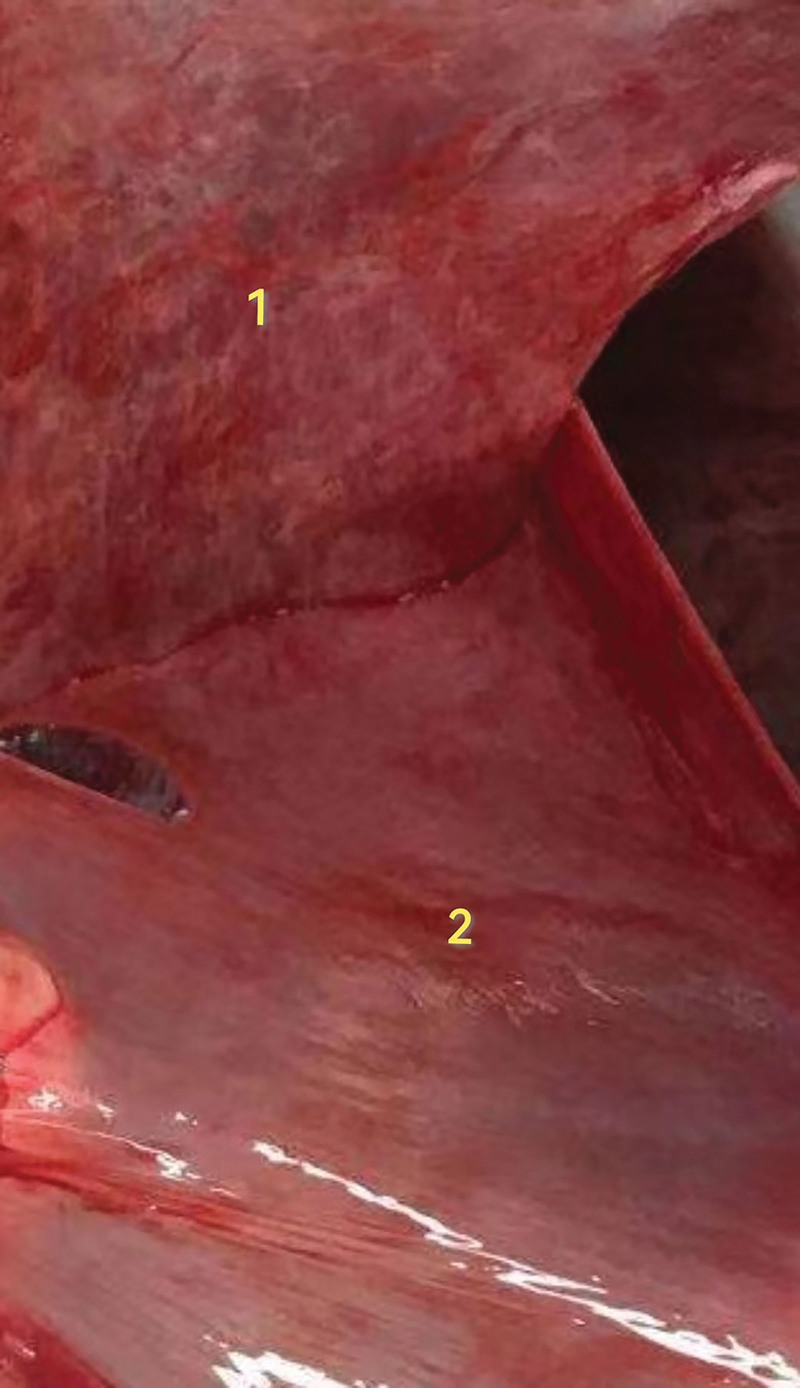
Fetal membranes consist of smooth chorion and amniotic membrane. (1: chorion; 2: amniotic membrane).

**Figure 2.**
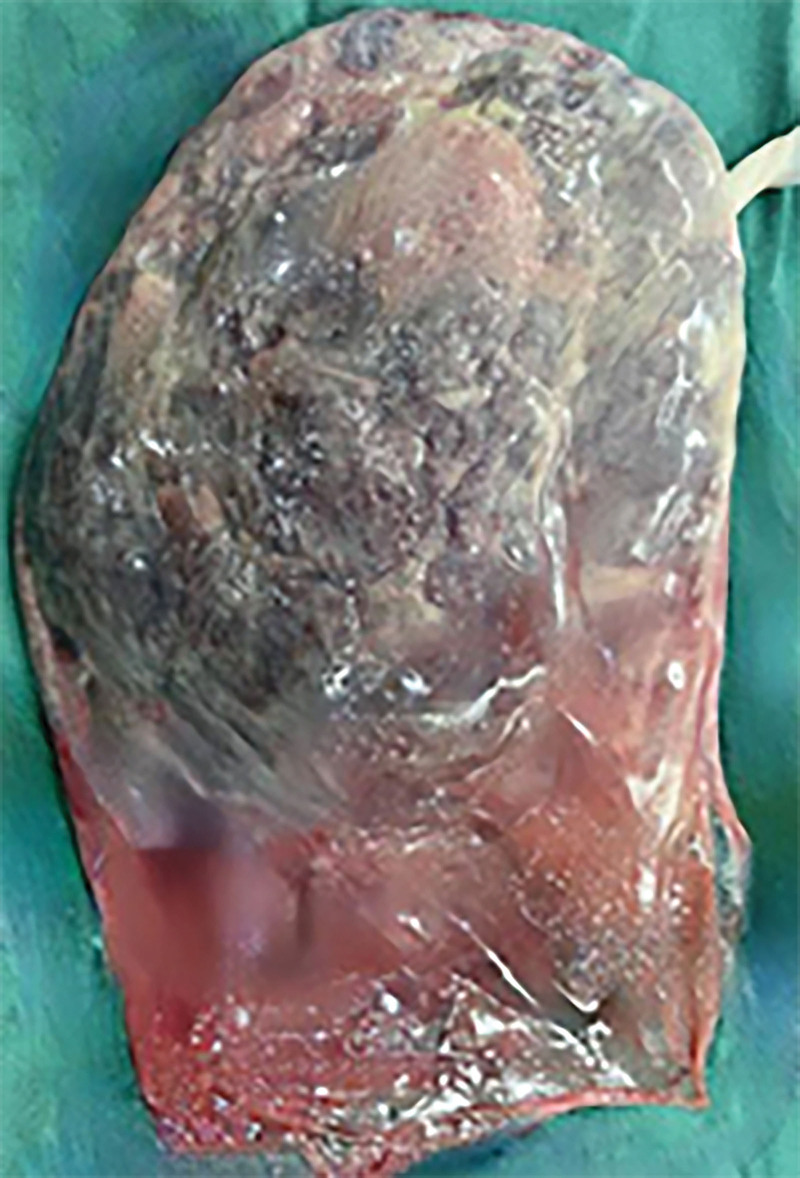
The membranes completely cover and extend beyond the placenta in singleton childbirth.

The fetal membranes consist of chorion laeve and amniotic membrane. The chorion laeve was formed by the gradually degenerated villi adjacent to the chorion laeve and decidua capsularis (the decidua overlying the blastocyst) due to lack of blood supply and nutrition. Amniotic membrane, the fetal part constituting the placenta, is the innermost layer of the placenta. It is translucent, smooth, and elastic without blood vessels, nerves and lymph, with a thickness of 0.02 to 0.05 mm^[3,4]^ (Fig. 1). In clinical practice, the fetal membrane in a single fetus is considered to be intact if the 2 layers, chorion laeve and amniotic membrane, are visible and can completely cover as well as extend over the placenta (Fig. 2).

**Figure 3.**
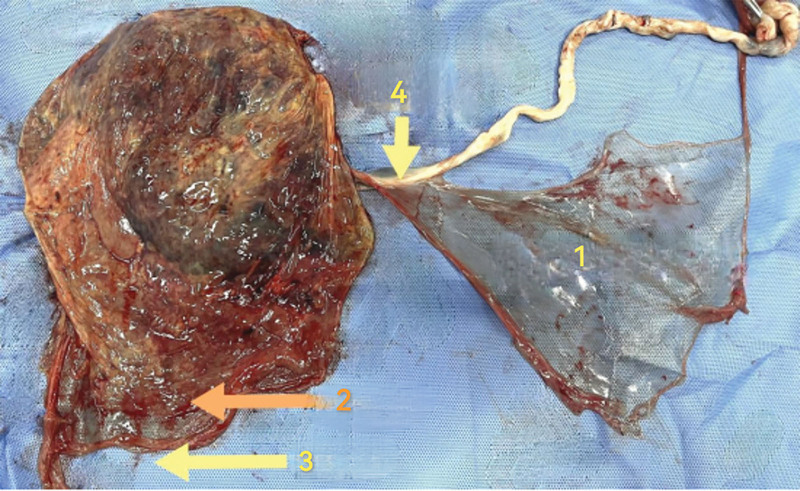
The connecting site of umbilical cord and layered thin amniotic membranes is extremely fragile and easy to break. (1: layered amniotic membrane (thin); 2: chorion; 3: amniotic membrane; 4: the connecting site of umbilical cord and fetal membrane is extremely fragile and easy to break).

The reasons why residual fetal membranes were not detected in this case were as follows: ① Risk cognition requires to be intensified: In this case, this was a precipitate labor with a short third stage of delivery, and thus there was a high risk of placental residual. Risk awareness requires to be enhanced. The patient was a multipara parturient whose birth canal was looser than that of the primipara. Therefore, the delivery speed of the placenta was difficult to control. There was no time left for the midwife to hold the placenta by both hands, rotated it in 1 direction and slowly pulled it out.^[4]^ However, if the fetal membrane was delivered under tension, it was easily partially ruptured, increasing the probability of residues. ② Lack of observation and thinking. As mentioned in the review of the relevant knowledge, the amniotic membrane was elastic with a certain thickness. If more careful observation is made in clinical practice, it can be found that the amniotic membrane with relatively thick texture and strong toughness can be further layered. The layered amniotic membrane gradually becomes thinner from the laceration of the fetal membrane to the site where the umbilical cord attached, until it is completely lacerated. It is relatively challenging to find the residue layered membrane during placental examination (Fig. 3). Considering that the placenta was delivered under tension, we could not exclude the possibility of the intrauterine residue of ring-shaped layered amniotic membrane distal to site where the umbilical cord attached. During placenta examination in our case, it was mistaken as being intact since a complete thin layer of amniotic membrane was observed. ③ The standard operating procedure needs to be perfected. After the delivery of the placenta, the placenta should be flattened out, and maternal placenta should be examined first to find if there is any missing of ruptured cotyledon.

The placenta is then lifted to check whether the fetal membrane is intact. The edge of the fetal surface of placenta is then examined for vascular rupture. Accessory placenta should be identified timely. There is no standard protocol on how to complete the examination of membranes. ④ Poor treatment measures. We only paid attention to vaginal hemorrhage after delivery. In the case of less vaginal hemorrhage, we rarely paid attention to the administration of uterotonic agents, and no uterine massage was provided to the patient. Lack of the above measures might lead to the failure in discharge of remaining fetal membrane in the uterine cavity.

## 4. Conclusions and lessons learned from this case

① Before placental residue is diagnosed after vaginal delivery, active management is recommended to promote spontaneous placental abruption. The commonly applied treatment methods include uterine contraction promotion, controlled umbilical cord traction and uterine massage.^[5]^ In particular, the controlled umbilical cord traction using “windmill technique” in the research by Hinkson et al can significantly reduce subsequent invasive operations.^[6]^ ② Strengthen learning of relative knowledge and the ability of active observation in clinical practice. Combine theory and practice so as to achieve the purpose of using theory to guide clinical practice and using clinical evidence to support theory; ③ In clinical practice, examination protocols should be created based on the principle of placental examination. Clinical standard and reference should be clearly illustrated which is easy to understand and memorize (such as video) (Table 1) so that standardized and effective placental examination can be conducted. ④ According to the disease evaluation framework, maternal condition should be comprehensively and effectively evaluated. Appropriate treatment measures should be applied timely and effectively applied to ensure the safety of parturient, decrease short-term and long-term complications, improve the quality of delivery, and reduce the potential dispute. ⑤ During placental examination, attention should be paid to whether there is delamination between amnion and chorion and missing part in layers to determine the integrity of fetal membrane. ⑥ The current standard operating procedure for examining the fetal membrane is that the two-layer fetal membranes should completely cover and exceed the edge of placenta. However, due to the differences in the size of placenta and uterine cavity of parturients, “exceeding” is not quantitatively defined and may also lead to fetal membrane residue, which requires further clinical research.

**Table 1:**
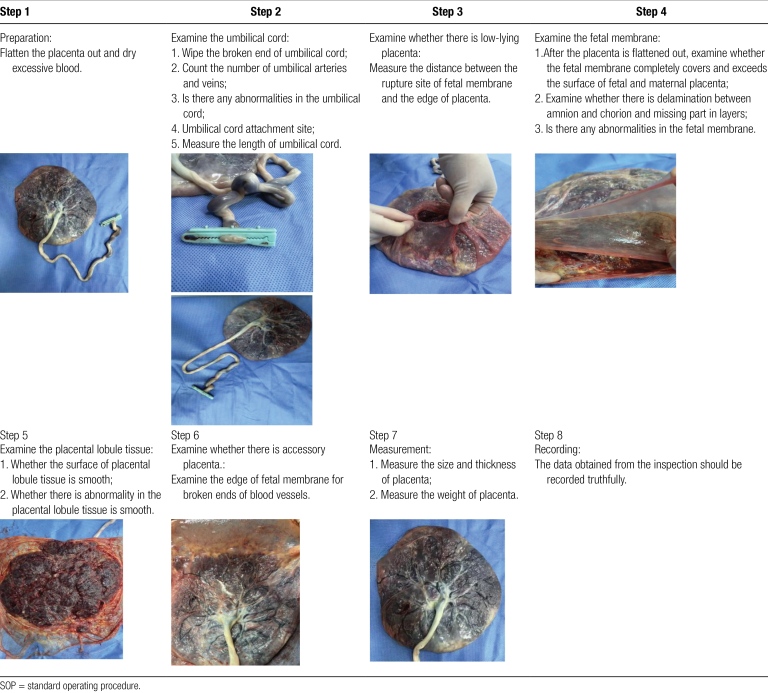
SOP for placental examination.

## Author contributions

**Conceptualization:** Yonghong Wang, Juan Li, Qiuyang He.

**Data curation:** Jianhua Ren.

**Formal analysis:** Jianhua Ren.

**Investigation:** Guoyu Wang.

**Methodology:** Yonghong Wang, Juan Li.

**Resources:** Guoyu Wang.

**Software:** Jianhua Ren.

**Supervision:** Guoyu Wang.

**Writing – original draft:** Yonghong Wang, Juan Li.

**Writing – review & editing:** Qiuyang He.
